# Home Range Use and Movement Patterns of Non-Native Feral Goats in a Tropical Island Montane Dry Landscape

**DOI:** 10.1371/journal.pone.0119231

**Published:** 2015-03-25

**Authors:** Mark W. Chynoweth, Christopher A. Lepczyk, Creighton M. Litton, Steven C. Hess, James R. Kellner, Susan Cordell

**Affiliations:** 1 Department of Natural Resources and Environmental Management, University of Hawai‘i at Mānoa, Honolulu, Hawai‘i, United States of America; 2 U.S. Geological Survey Pacific Island Ecosystems Research Center, Hawai‘i National Park, Hawai‘i, United States of America; 3 Department of Ecology and Evolutionary Biology, Brown University, Providence, Rhode Island, United States of America; 4 U.S. Department of Agriculture Forest Service, Institute of Pacific Islands Forestry, Hilo, Hawai‘i, United States of America; University of New England, AUSTRALIA

## Abstract

Advances in wildlife telemetry and remote sensing technology facilitate studies of broad-scale movements of ungulates in relation to phenological shifts in vegetation. In tropical island dry landscapes, home range use and movements of non-native feral goats (*Capra hircus*) are largely unknown, yet this information is important to help guide the conservation and restoration of some of the world’s most critically endangered ecosystems. We hypothesized that feral goats would respond to resource pulses in vegetation by traveling to areas of recent green-up. To address this hypothesis, we fitted six male and seven female feral goats with Global Positioning System (GPS) collars equipped with an Argos satellite upload link to examine goat movements in relation to the plant phenology using the Normalized Difference Vegetation Index (NDVI). Movement patterns of 50% of males and 40% of females suggested conditional movement between non-overlapping home ranges throughout the year. A shift in NDVI values corresponded with movement between primary and secondary ranges of goats that exhibited long-distance movement, suggesting that vegetation phenology as captured by NDVI is a good indicator of the habitat and movement patterns of feral goats in tropical island dry landscapes. In the context of conservation and restoration of tropical island landscapes, the results of our study identify how non-native feral goats use resources across a broad landscape to sustain their populations and facilitate invasion of native plant communities.

## Introduction

Studies of animal movement include a broad range of methods to understand how organisms interact with the surrounding environment [1,2]. Movements can range from fine scale observations of animal behavior to broad-scale migrations across landscapes. Understanding the major drivers of animal-movement patterns can help to directly manage species and both directly and indirectly address conservation issues at a variety of scales, including in systems where animals are non-native. Key external driving factors governing movement of large mammals can be identified using geographic information systems (GIS) and remotely sensed data to quantify vegetation composition, structure, and dynamics [3,4]. To understand how external factors influence movement, observed locations of individuals or populations over time can be used to estimate home ranges and broader movement patterns. These broad scale movement patterns are not only important in ecological processes, such as habitat fragmentation and biological invasions [6], but are also important in the context of management.

Although ultimate causes for movement, such as competition for mates and inbreeding avoidance, can be a selective advantage [7], proximate causes for movement are often related to resource availability and inter-patch movement [8]. In some ecosystems, particularly those in dry areas or with limited resources, phenological events (i.e. vegetation green-up) represent a resource pulse, or a high intensity, infrequent event of increased resource availability for herbivores [9]. These variations in vegetation resources are often the result of precipitation events that occur as pulses in dry landscapes [10,11]. Pulse precipitation events are hypothesized to influence phenological shifts in vegetation and hence drive movement of large ungulates [12,13]. For example, red deer (*Cervus elaphus*) have been observed to follow this ‘green wave’ of vegetation to gain access to early plant phenology [14]. The combination of remotely sensed phenology and animal movement datasets has only recently allowed ecologists to test the pulse precipitation hypothesis.

Ungulates inhabiting grasslands have shown strong responses to temporal changes in above ground net primary productivity [15]. Net primary productivity is often quantified using a variety of vegetation indices generated from global remote sensing datasets. In particular, the Normalized Difference Vegetation Index (NDVI) has shown a strong correlation with phenological characteristics [16], and has been recognized as a valuable tool in coupling net primary productivity to the behavioral ecology of animals [4], such as analyzing ungulate movement patterns in multiple ecosystems [17–20].

The difficulty and expense of monitoring large mammals over long periods has often prevented the acquisition of empirical data documenting fine scale movement of animals across broad landscapes. The use of Global Positioning System (GPS) wildlife collars allows the collection of high resolution spatiotemporal data, providing a detailed examination of home range use by large mammals [21]. By combining these high resolution GPS data with remotely sensed imagery, home range, movement, and migration events can be examined at broad scales, and hypotheses related to movement and resource availability can be tested [3]. Specifically, NDVI data can be used to characterize broad scale movement patterns in response to phenological shifts across ungulate home ranges [14,19,22,23], providing an understanding of movement that can be applied to the conservation and restoration of ecosystems and landscapes.

On island landscapes, non-native feral goats (*Capra hircus*) have a large effect where they have invaded and represent a significant threat to conservation and restoration of native ecosystems [24]. Feral goats have degraded native ecosystems on islands throughout the Pacific Ocean and have been particularly deleterious in Hawaiian montane dry landscapes since their introduction in the late eighteenth century [25]. As an extreme generalist, feral goats modify the landscape directly through consumption and trampling of both native and non-native plants which can lead to indirect effects such as modification of ecosystem structure and function. These effects are intensified in ecosystems, such as the Hawaiian Islands, that have evolved in the absence of large mammalian herbivores. Although fenced exclosure studies have documented the effects of ungulates on native Hawaiian ecosystems [26,27], understanding home range, space use, and movement patterns with the aid of next generation tools (e.g., GPS and remote sensing) will help prioritize landscape conservation and restoration efforts in montane dry landscapes. Hence, the objectives of this study were to use GPS collars and remotely sensed data in a large tropical dry landscape to: 1) estimate home range size; and 2) determine whether the movements of feral goats are related to pulses in vegetation resources. Previous work on the movement of large herbivores suggests that at least some species respond to vegetation phenology by moving to areas of increased primary productivity [4,22,23]. Based on this previous research, we hypothesized that feral goats would respond to resource pulses in vegetation by traveling to areas of recent green-up. To test these hypotheses, we deployed GPS collars on feral goats to quantify home range size and determine if movement patterns relate to patterns in vegetation phenology or greenness.

## Materials and Methods

### Study Area

**Fig 1:**
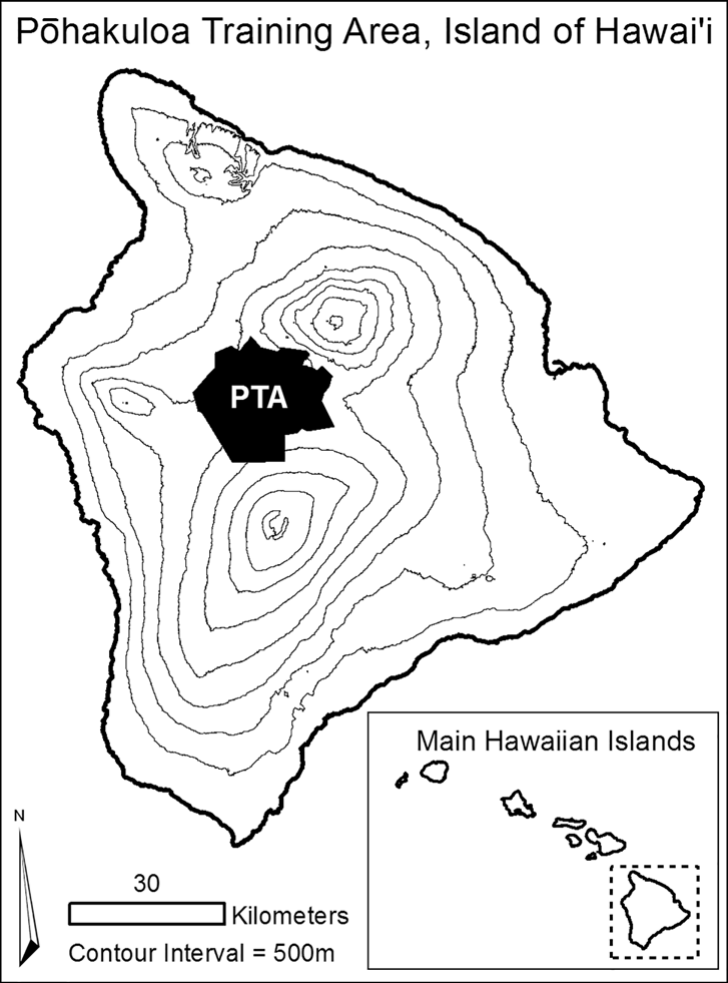
Location of the study area: Pōhakuloa Training Area on Hawai‘i Island (PTA). The PTA is a tropical island montane dry landscape that lies at approximately 1900 meters in the saddle of three volcanoes. We captured and collared 13 non-native feral goats in the northern section, 2010–2011.

We conducted a telemetry study on feral goats between July 2010 and July 2011 in the Pōhakuloa Training Area (PTA) on Hawai‘i Island (19°45′36″N 155°33′13″W; Fig. 1). Permission to conduct research at PTA was provided by the Environmental Division, Directorate of Public Works, U.S. Army Garrison-Pohakuloa. PTA is a 438 km^2^ active military installation lying in the saddle of three volcanoes, Mauna Kea (4205 m), Mauna Loa (4169 m), and Hualālai (2521 m), which covers both the Koppen temperate climate zones Cfb (maritime temperate climates: continuously wet warm temperate) and Csb (dry-summer subtropical: summer-dry warm temperate). PTA has high climatic variability, with temperatures ranging from 10 to 22°C during at least 4 months of the year. Seventy percent of the annual rainfall (561 mm annual average) typically occurs between November and March, and the driest summer month (August) receives <30mm of rainfall in the Csb climate [28]. PTA is comprised of a complex mosaic of plant communities that have resulted from spatial variability in substrate type and age, and subsequent soil development. Sections of Hawai‘i’s last remaining tropical montane dry forests are present in PTA, including the following major plant communities: *Metrosideros* woodland, *Dodonaea* shrubland, and *Myoporum-Sophora* woodland, as well as native *Eragrostis* sp. and nonnative *Cenchrus* sp. grasslands [29]. Although feral goats occur across five of the eight largest Hawaiian Islands in virtually every habitat type, a particularly high density of these animals occupy dry montane landscapes such as PTA. No quantitative data exist on feral goat abundance at PTA, but a 2009 animal drive forced approximately 1800 feral goats out of a fenced management unit of 21.3 km² [30], which equates to a density of 1.9 animals ha^-1^. Feral goats are actively hunted at PTA, with variable hunter access depending on military training activity and local regulations.

### Feral Goat Capture

On July 2^nd^, 2010, 12 adult (>18 months old; [31]) feral goats were captured by net gun using an MD 500D helicopter platform in the northern portion of PTA. Potential capture locations were limited due to extensive fenced exclosures and a large off-limits ordnance area with active artillery training. To achieve a representative sample, individuals were selected based on spatial location (i.e. individuals from 12 distinct herds or groups to maximize collar efficiency), as well as sex and age classes. One collar was redeployed after the initial mortality event; in total 13 adult feral goats were captured over the course of this study (6 males, 7 females). Collars with >250 days of data were used in movement pattern analysis (*n* = 11). Capture and handling methods were approved by the University of Hawai‘i at Mānoa Institutional Animal Care and Use Committee (Protocol #10–868).

### Feral Goat Monitoring

GPS Argos wildlife collars (model GPS7000SA, Lotek Wireless, Newmarket, Ontario, Canada) weighing approximately 450 grams (< 2% body weight) were attached to the animals after aging and health assessment. GPS-collars were equipped with two separate transmitters: (*i*) a VHF transmitter for locating animals for field observations, and (*ii*) an Argos transmitter for remote data download via satellite. Collars were programmed to log a GPS location and ambient temperature every two hours for one year, and download location data via the Argos network once every five days. Logging fixes every two hours allowed for the maximum amount of data (shortest interval) to be collected over the one-year study period. Animals were relocated using the VHF transmitter throughout the summer of 2010 to confirm that individuals were in separate herds and to ensure that collars were not impeding movement.

Data were collected from collars in two ways. First, data were downloaded remotely from individual collars every five days via the Argos network due to the high risk of equipment loss, failure due to harvest by hunters, or mortality at locations with no VHF coverage (e.g., cave or lava tube) or where unexploded ordnance restricted access. Second, collars stored all data for downloading upon final retrieval when a pre-programmed mechanism caused collars to drop off animals after 365 days.

Animal locations were input to ArcGIS 9.3.1/10.0 GIS (Environmental System Research Institute Inc., Redlands, CA, USA). Only location fixes with a three dimensional fix and low Positional Dilution of Position value (PDOP < 3) were included in datasets for analysis [32]. Argos location data were also collected during remote downloads but were not used in further analyses due to inaccuracy and infrequency of data collection [33]. A total of 31,108 GPS fixes were collected from July 2010 to July 2011. Nine collars lasted the full study period, while two collars failed for unknown reasons, and two mortality events occurred.

### Feral Goat Home Range Analysis

Utilization distribution (UD), home range area, and core-use area estimates were calculated using adaptive-kernel density estimators [34] with the Home Range Tools (HRT) Analysis Extension in ArcMap 9.3 [35]. Home range estimates were generated with an ad hoc smoothing parameter (h_*ad hoc*_) using the smallest increment of the reference bandwidth (h_*ref*_) that provided a contiguous 95% kernel home range (i.e. h = 0.5 × h_*ref*_, 0.6 × h_*ref*_,… h_*ref*_–J. Kie, pers. comm.). The number of points used to generate annual and seasonal utilization distributions ranged from 381 to 3,033, providing robust estimates of kernel density [36]. Home range estimates provide a 95% utilization distribution, a 95% home range, and a 50% core-use area for each feral goat at a 5×5 m resolution.

### Feral Goat Interaction Analysis

Interactions between collared individuals were estimated using two methods. First, congruence of 95% fixed kernel UDs was measured for overlapping individuals by using the UD_1_(*x,y*) Utilization Distribution Overlapping Index (UDOI) developed by Fieberg and Kochanny (2005). UDOI index values range from 0.0 (no overlap) to 2.0 (complete overlap). UDOI values <1 indicate less congruence in UD than would be expected from overlapping distributions, whereas values >1 indicate greater congruence in overlapping UD than would be expected. UDOI values were calculated in R (R Development Core Team, 2011) using the *adehabitat* extension [38].

Second, association between individuals was estimated based on distance between each individual location, because association or segregation between individuals may occur at a finer scale than UDOI can detect. Influences within home ranges, such as social or habitat factors, may cause segregation. To address this, the software package ASSOC1 [39] was used to investigate the spatiotemporal association of individual collared animals at the 24 hour temporal scale. ASSOC1 uses association matrices to determine the amount of time each individual feral goat was located within a user-defined spatial threshold of each collared individual. Given that each individual represents a sampling unit, this analysis assured that pseudo-replication [40] was avoided in further analyses, and allowed examination of social associations between collared individuals [41]. Spatial and temporal parameters were determined based on field observations of herd dynamics and repeated model runs. A spatial threshold of 400 m and temporal threshold of 75%, meaning individuals had to be within 400 m for 75% of the location estimates to be considered associated, captured major group interactions. Results are reported as percent of points that are considered associated.

### Feral Goat Movement Pattern Analysis

To identify long-distance movement events, each animal’s movement patterns were examined for unidirectional movements over a long distance (>diameter of home range) and short period of time (<2 days). The harmonic mean of animal locations was used to determine the geographic center of non-overlapping home ranges [42]. Non-overlapping ranges were termed primary and secondary ranges to distinguish between the two areas used by feral goats, but these terms are not meant to suggest any difference in importance between ranges. Linear distances between activity centers of non-overlapping home ranges were measured in GIS [43].

### Phenological Monitoring

We used NDVI to quantify temporal changes in vegetation phenology and to link this to long-distance movement events of feral goats. NDVI has been shown to respond to several different environmental variables, including precipitation events [16,44]. In Hawaiian dry landscapes, as pulse precipitation events occur, photosynthetic activity associated with green-up events can be detected with remotely sensed imagery as specific changes in spectral wavelengths [45]. To obtain NDVI values, data were calculated from the Moderate Resolution Imaging Spectrometer sensor (MODIS, Raytheon Co., Waltham, MA USA). MODIS sensors are part of NASA’s Earth Observing System (EOS) program to observe spatial and temporal variations in vegetation with a coordinated set of polar orbiting satellites. Daily global images are used to estimate vegetation indices (e.g., NDVI) and provide a measure of vegetation greenness based on the ratio between near-infrared and visible reflectance (i.e., (NIR-VIS)/(NIR+VIS)). NDVI values range from -1.0 to +1.0, with negative values indicating surfaces with little or no vegetation (i.e. barren ground, water, rock) and positive values indicating increasing amount of green vegetation.

For calculation of NDVI, we used 16-day composite MODIS Vegetation Index NDVI data sets (MOD13Q1 product) with 250 m pixel resolution. Data were acquired through NASA’s EOS Data and Information System (http://reverb.echo.nasa.gov/reverb/; tile number: H03V07). Using 24 images, a time series was created from 26 June 2010 to 26 June 2011. Downloaded images were only available in the Sinusoidal Universal Transverse Mercator projection Zone 5 on the North American Datum 83 projection. We used the MODIS Reprojection Tool (NASA Land Processes Distributed Active Archive Center) to project the data into the Universal Transverse Mercator projection Zone 5 on the North American Datum 1983.

Reprojected images were then imported into ArcMap 10.0 to calculate mean NDVI of each home range for each time interval. Following the methods of Leimgruber et al. (2000) and Ito et al. (2006), mean NDVI values of annual ranges were subtracted from every time interval to obtain an index of relative quality of different ranges within annual ranges. A Wilcoxon signed rank test was used to examine the differences in relative NDVI values between primary and secondary ranges [23].

### Statistical Analysis

Individual mean NDVI values were used for home range comparisons between sexes and between primary and secondary ranges of individuals that demonstrated long-distance movements. All means are reported with associated standard errors. For home range comparisons, long-distance movement periods, and movement distances, a two-way Welch’s *t-*test was used to account for small sample sizes and heterogeneous variances. Two-tailed significance values were reported as the hypotheses were two-sided, and significance was assessed at α = 0.05. To compare NDVI rank values of repeated measures of primary and secondary ranges, a Wilcoxon signed-rank was used to test differences in mean ranks. One-tailed significance values were reported as the hypotheses were one-sided, and significance was assessed at α = 0.05. All statistical analysis were conducted in R: A language and environment for statistical computing 2.13.2 [37].

## Results

### Feral Goat Home Ranges

Home ranges for both sexes of feral goats spanned from 3.4 to 60.0 km^2^ (Table 1). Male annual home range was 40.0 ± 7.9 km^2^ (range 5.9–60.0 km^2^) compared to 13.3 ± 4.7 km^2^ (range 3.4–27.7 km^2^) for females. Similarly, mean annual 50% core use area for males was 8.0 ± 1.9 km^2^ (range 1.1–15.1 km^2^) compared to 2.9 ± 1.1 km^2^ (range 0.8–7.8 km^2^) for females. The 95% annual home ranges were significantly larger for males than females (*t* = 2.65, *d*.*f*. = 8.67, *P* = 0.027), but the annual 50% core use areas did not differ statistically between sexes (*t* = 2.13, *d*.*f*. = 8.69, *P* = 0.063).

**Table 1:** Adaptive-kernel density estimates with *href* for the smoothing parameter of annual home range and core-use area of 13 feral goats in Pōhakuloa Training Area on Hawai‘i Island, 2010–2011.

Goat ID	Monitoring period (#days)	# of points	95% Area (km^2^)	50% Core-use Area (km^2^)
F1	299	2554	27.7	6.4
F2	363	2519	7	1.3
F3	309	2512	34.7	7.8
F4	363	2990	7.1	1.3
F5	46	381	3.4	0.8
F6	127	636	7.7	1.7
F7	363	2513	5.8	0.9
M1	363	2870	43.3	7.5
M2	363	2568	60	15.1
M3	363	2622	53.8	9.8
M4	363	3033	5.9	1.1
M5	363	2985	44.9	8.5
M6	363	2925	31.9	6.2
**Mean male 95% area**			**40.0 ± 7.9 km** ^**2**^	
**Mean female 95% area**			**13.3 ± 4.7 km** ^**2**^	
**Mean male 50% area**			**8.0 ± 1.9 km** ^**2**^	
**Mean female 50% area**			**2.9 ± 1.1 km** ^**2**^	

### Feral Goat Interaction Analysis

The UDOI index of UD overlap indicated that most feral goats showed less overlap than would be expected from overlapping distributions at the 95% and 50% contour levels (S1 Fig.). Mean UDOI values of 95% UDs for males and females were 0.176 ± 0.063 and 0.334 ± 0.058, respectively. For 50% UDs, mean UDOI values for males and females were 0.009 ± 0.005 and 0.023 ± 0.005, respectively. On average, males showed less overlap than females. Daily mean social association (proportion of points within 400 m) was 5.9 ± 0.5% during the day and 12.7 ± 0.1% at night (S2 Fig.). Collared animals had higher levels of association overnight in comparison to daytime, suggesting fission of herds during the day and fusion of herds at night.

### Feral Goat Movement Patterns

**Fig 2:**
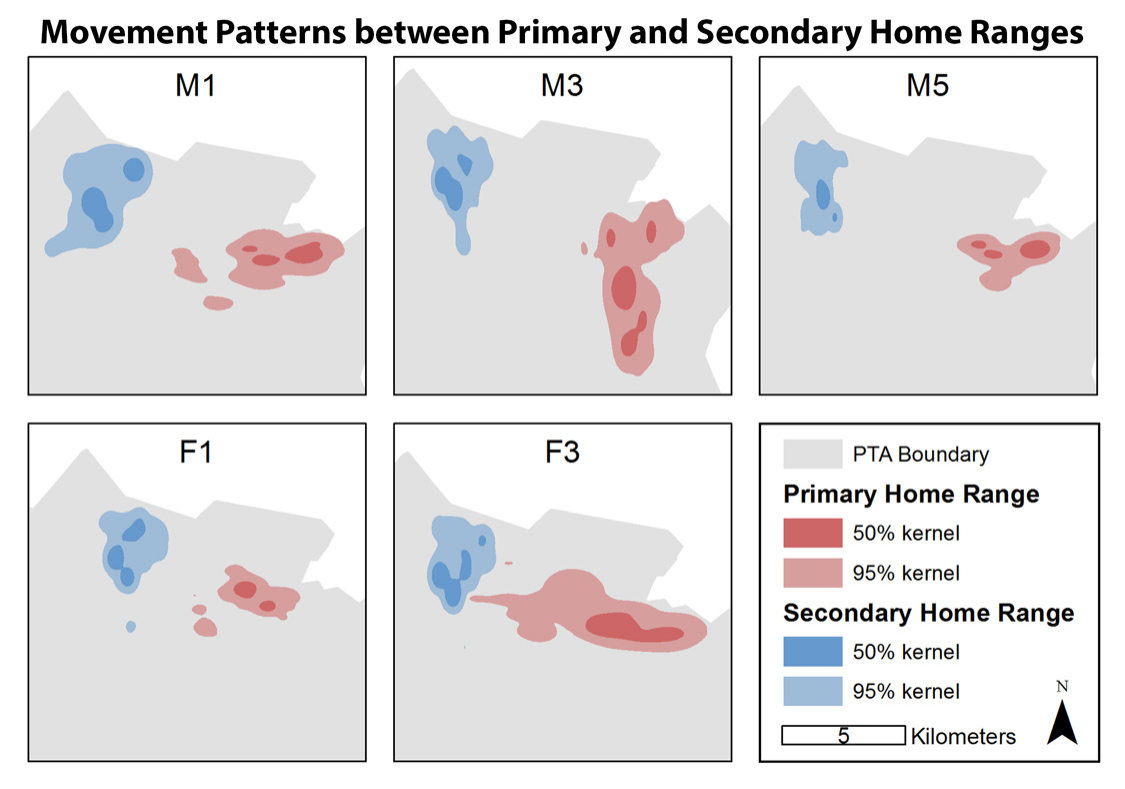
Primary and secondary home ranges of long-distance movement feral goats. Adaptive kernel home ranges for 5 non-native feral goats that moved between non-overlapping home ranges in Pōhakuloa Training Area on Hawai‘i Island, 2010–2011. Red areas indicate 50% (dark red) and 95% (light red) primary ranges and blue areas indicate 50% (dark blue) and 95% (light blue) primary ranges. All individuals moved WNW to the only region of the study area that experienced significant vegetation green-up.

Among all feral goats, 5 out of 11 individuals had 7 long-distance movements (Fig. 2). The remaining 6 individuals demonstrated limited annual variation in home range size and no long distance movement events. Of the five individuals that demonstrated long distance movement, mean movement distance was 7.71 km (SE = 0.63 km). While movements to secondary home ranges usually took place over a one-day period, departure date varied slightly throughout the year. There was no difference (*t* = 0.02, *d*.*f*. = 9.82, *P* = 0.99) between primary ($\overset{-}{X}$ = 11.69 km^2^, SE = 2.01) and secondary ($\overset{-}{X}$ = 11.64 km^2^, SE = 2.31) home range sizes of dispersing individuals (S1 Table).

**Fig 3:**
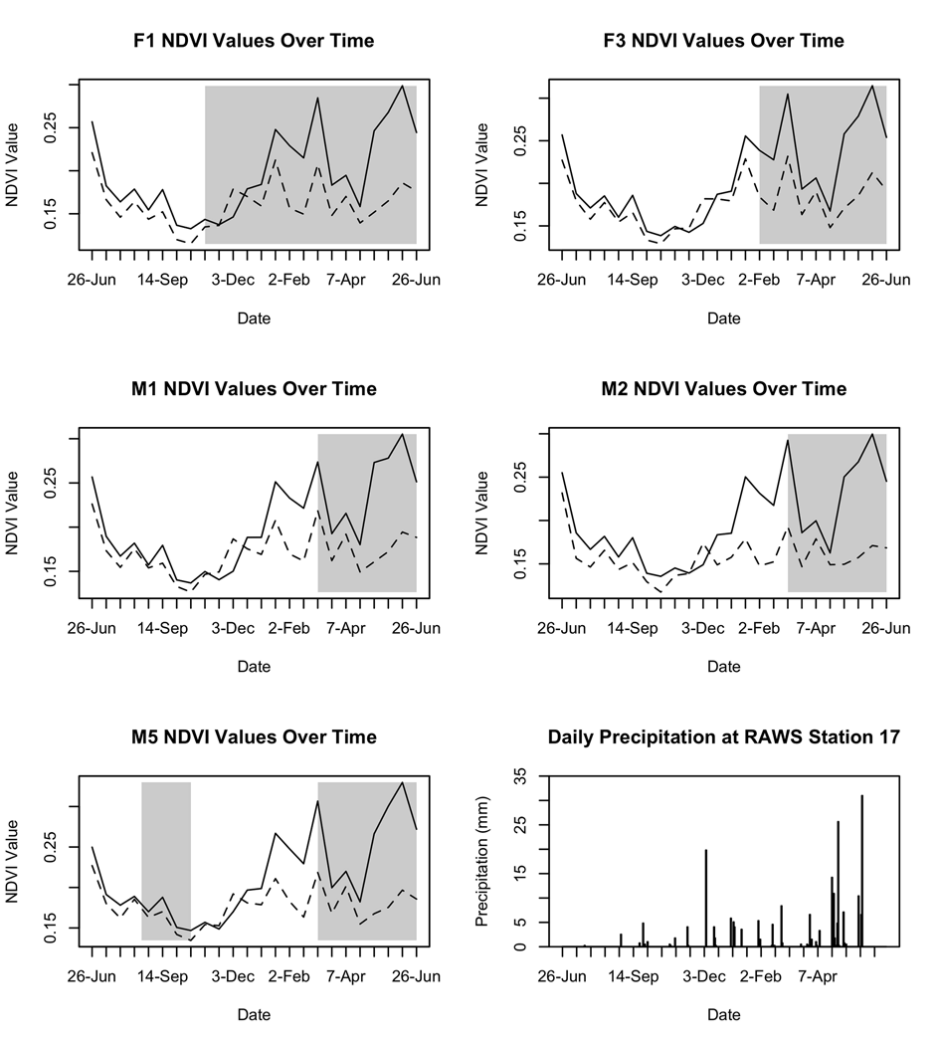
Phenology of feral goat movement. A comparison of mean Normalized Difference Vegetation Index (NDVI) values in primary and secondary ranges of non-native feral goats in Pōhakuloa Training Area on Hawai‘i Island, 2010–2011. White regions of the graph represent time when individuals are located in Primary Ranges and shaded regions represent time when individuals are located in Secondary Ranges. The mean NDVI value of individual Primary and Secondary ranges are represented by dotted and solid lines, respectively.

Mean NDVI values in primary and secondary home ranges showed similar trends over one year. Both primary and secondary ranges showed an increase in NDVI during the second half of the study associated with increases in the frequency and intensity of precipitation events (Fig. 3). However, a greater increase in NDVI occurred in secondary vs. primary home ranges of all dispersing individuals. Specifically, four out of five individuals dispersed to a secondary range that had significantly higher NDVI values compared to their primary ranges (Table 2).

**Table 2:** One-tailed probabilities for differences in relative NDVI values between primary and secondary ranges of feral goats in Pōhakuloa Training Area on Hawai‘i Island, 2010–2011.

Goat ID	*z-score*	V	*p*	Higher NDVI range
F1	-0.14	17	0.945	n.s.
F3	-2.7011	1	0.008	Secondary
M1	-2.5205	0	0.016	Secondary
M2	-2.2404	2	0.046	Secondary
M5	-2.4006	6	0.028	Secondary

## Discussion

Annual home ranges demonstrated extensive two-dimensional overlap, but analysis of herd association suggests that feral goats exhibited daytime herd fission and nighttime fusion. Nearly half of the individuals being tracked demonstrated long-distance movement behavior. Based on NDVI values of primary and secondary home ranges of dispersing individuals calculated with kernel density estimators, results support the hypothesis that feral goats travelled to areas of recent vegetation green-up following pulse precipitation events. Some limitations existed in our study; in particular, how human activity on this active military base may influence feral goat movement. However, the patterns that we observed suggest that the NDVI is a good indicator of habitat and movement patterns of feral goats in tropical island dry landscapes.

### Feral Goat Home Range

Our estimates for the sizes of home ranges for feral goats in Hawai‘i are within the range of estimates (0.4–246.5 km^2^) for other dryland habitats [46,47]. In comparison to these other studies, home ranges in our study encompassed a similar amount of space, but 50% core use areas were substantially smaller than annual ranges. This difference suggests that feral goats used space non-randomly, returning to multiple core use areas within annual ranges. Based on collar data and field observations, core areas were bedding grounds used on a nightly basis. These bedding grounds often included areas of high topographic variability with high lookout points, a valuable resource for predator detection and avoidance [24].

Annual home range estimates were highly variable between individuals and sex (Table 1). For individuals demonstrating long-distance movement movements, mean annual estimates included primary and secondary ranges, which may have overestimated home range size. The differences between male and female home ranges relating to activity budgets is common in many ungulate species [48]. This difference between sexes could be attributed to sexual segregation of herds, which was observed throughout the study period. Sexual segregation did not appear to be a function of habitat preference. Instead, four principal hypotheses potentially explain segregation in feral goats: predation, forage quality, social preferences, and activity patterning [49]. Depredation by feral dogs (*Canis familiaris*) is likely, but its extent and simultaneous interaction with the three remaining hypotheses are unknown, making its effect on sexual segregation difficult to disentangle.

### Feral Goat Interaction

Feral goats are highly social animals and this influences range size and movement patterns across the landscape [50]. Observations of herd size and composition at PTA are structurally similar to feral goat populations on other islands [47,51]. Two-dimensional overlap suggests that animals are sharing large portions of their home range. However, based on the UDOI index, animals occupying overlapping home ranges had multiple core areas throughout the range that were used at different times during the year. Sexual segregation of ungulates is common, and it is important to note that juvenile feral goats and pregnant females were observed regularly, evidence of year-round breeding that occurs in other island systems [52]. Our analysis of association of collared individuals using the ASSOC1 software package suggests that, concordant with other studies on herd dynamics of feral goats [51,53,54], collared individuals are near other collared individuals more frequently during nocturnal hours and less frequently during diurnal hours.

### Feral Goat Movement Patterns

Both males and females demonstrated long-distance movement, and each movement was unidirectional. With the exception of two individuals, feral goats dispersed at different times throughout the year. Each movement was a shift from the eastern section (primary range) to northwestern section (secondary range) of PTA, and each long-distance movement was consistent with the hypothesis that feral goats respond to intra-seasonal vegetation dynamics on small temporal scales by traveling to areas of recent vegetation green-up. Mean secondary home range size was slightly smaller than mean primary home range size, suggesting that increased resource availability associated with vegetation green-up requires less space-use by feral goats. While the difference in area between primary and secondary home ranges had no statistically significant difference, there may be an ecologically significant difference undetected due to small sample size.

Feral goats in this study did not exhibit movement patterns consistent with animal migration. Although migration is sometimes defined as movement from one spatial unit to another [55], it is more appropriately classified to include an animal’s return to a primary range [5]. Four of the five animals that demonstrated long-distance movement exhibited one single movement from primary to secondary ranges, suggesting dispersal-like behavior but not migration. One individual made three long-distance movements throughout the year between primary and secondary home ranges. However, the time frame of this study and lifespan of collars did not provide replicates for seasons and was not sufficient to capture annual movement patterns of other individuals that may have displayed this behavior.

Six animals (three females and three males) did not move from a primary home range. However, four of those six animals resided year-round in or near the secondary range of animals that demonstrated long-distance movement. The primary range of these animals experienced the same NDVI patterns exhibited by secondary ranges of dispersing animals, suggesting that available resources increased in the primary range of feral goats, which would make long-distance movement undesirable. NDVI values were examined throughout the study area for green-up events, and few areas experienced a deviation of 100% from the mean NDVI values. The secondary home ranges of animals that demonstrated long-distance movement, and the primary ranges of 66% of non-dispersing animals were the only large areas that experienced substantial green-up events in the study area during collar deployment. During the 12 months of this study, weather stations within the primary study area received record low levels of precipitation (218.4mm) (561.2mm mean annual precipitation; [56]). Rainfall in 2009, 2010, and 2011 was 67.8%, 46.2%, and 65.7% of the long-term annual mean rainfall at 21, 34, and 31 climate stations, respectively, on Hawai‘i Island (National Weather Service, Honolulu). Drought severity clearly limited the frequency of green-up events during this study.

Our results suggest that long-distance movements by non-native feral goats in dry landscapes on tropical islands are spatially and temporally complex. Other factors that were not quantified in this study (e.g., herd dynamics, social structure) have been observed to influence the conditional movements of non-native feral goats in other study areas [47]. Our data shows that several collared individuals interacted on a semi-regular basis, demonstrating the fission-fusion pattern of herd dynamics evident in other studies [49]. Reproductive cycles, agonistic behavior, and density dependence are also examples of other factors not addressed in this study that may affect home ranges and movement [47,50]. Collectively, these same factors may have influenced the lack of movements among six individuals that remained stationary throughout the year.

Several factors associated with military training may have also limited the movement of the animals themselves. Feral goats may have avoided areas of human disturbance including structures, a gravel pit mine, and intermittent high-volume vehicular traffic. In addition, large fenced exclosures prevented the movement of animals into certain areas, which were incorporated into spatial analyses by masking these fenced areas during home range estimation. However, military training and other human activities were not available for assessment as a temporal factor influencing animal movement.

Based on our findings, strong evidence exists that feral goats move to areas of high NDVI values following pulse precipitation events in dry montane landscapes on tropical islands. Movement patterns of collared feral goats in PTA suggest neither nomadic behavior nor migration. Further research over a longer observational period (>1 year) would help determine if the movement patterns observed in this study are the result of ultimate or proximate causation. Results presented here contribute to a growing field of research in movement ecology that combines GPS telemetry data with remotely sensed phenological data to test hypotheses of herbivore movement in response to pulses in primary productivity. Although seasonality in the tropics is not as pronounced as temperate regions, PTA is a dry system that is characterized by both low and variable precipitation. These conditions occur in dry ecosystems throughout the world and offer important implications for conservation and management beyond just Pacific Islands.

## Supporting Information

S1 Fig The utilization distribution overlap index (UDOI) between annual home ranges of non-native feral goats in Pōhakuloa Training Area on Hawai‘i Island, 2010–2011. Individual home range overlap is compared to all other goats in the study. Overlap index values are presented for 95% UDs (graph A) and 50% UDs (graph B).
(TIF)

S2 Fig Mean daily association between all individual collared feral goats in Pōhakuloa Training Area on Hawai‘i Island, 2010–201l. Association is calculated hourly based on each location estimate. Spatial threshold: 400 m, temporal threshold: 75%. Percent refers to percent of total fixes that were within 400 m 75% of the total time.
(TIF)

S1 Table Adaptive-kernel density estimates with *href* for the smoothing parameter of primary and secondary home range and core-use area of 5 feral goats in Pōhakuloa Training Area on Hawai‘i Island, 2010–2011. (DOCX)
