# Supplementary figures and images for: Home Range Use and Movement Patterns of Non-Native Feral Goats in a Tropical Island Montane Dry Landscape
Source: PLoS One. 2015 Mar 25;10(3):e0119231. doi: 10.1371/journal.pone.0119231 (PMC4373820; doi:10.1371/journal.pone.0119231)

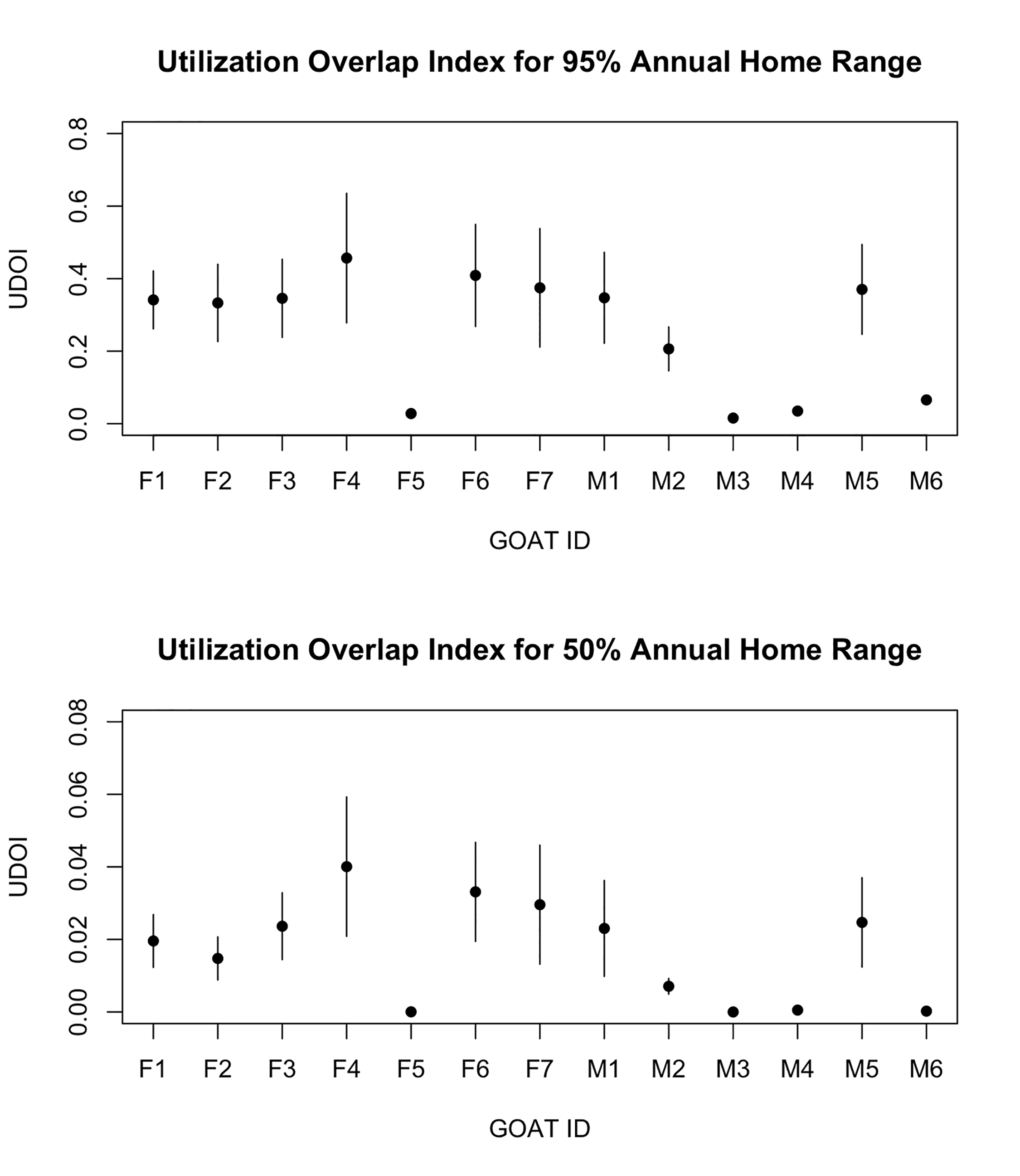

Supplement: S1 Fig — Individual home range overlap is compared to all other goats in the study. Overlap index values are presented for 95% UDs (graph A) and 50% UDs (graph B). (TIF) [file pone.0119231.s001.tif]

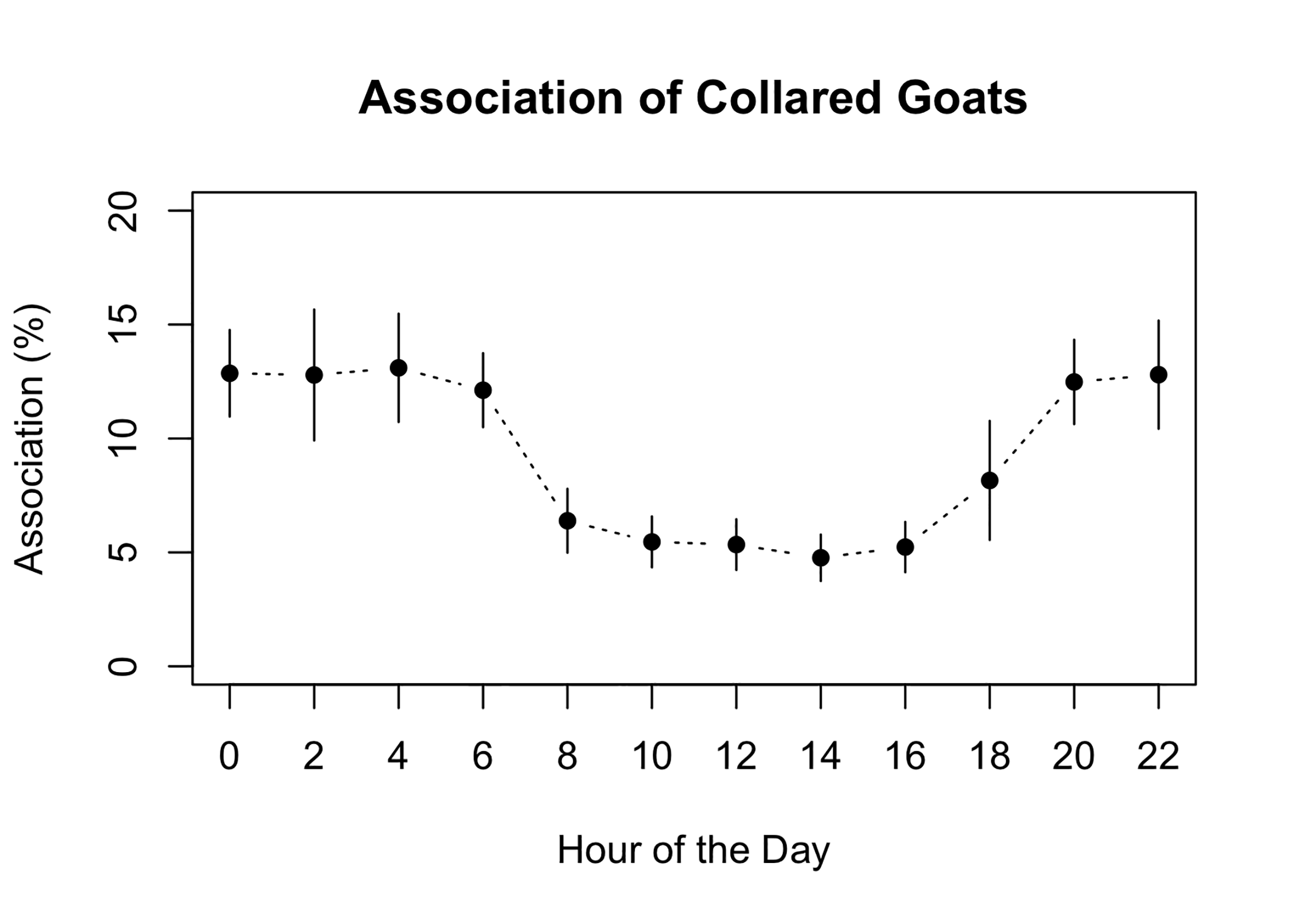

Supplement: S2 Fig — Association is calculated hourly based on each location estimate. Spatial threshold: 400 m, temporal threshold: 75%. Percent refers to percent of total fixes that were within 400 m 75% of the total time. (TIF) [file pone.0119231.s002.tif]
