# Supplementary material for: Home Range Use and Movement Patterns of Non-Native Feral Goats in a Tropical Island Montane Dry Landscape
Source: PLoS One. 2015 Mar 25;10(3):e0119231. doi: 10.1371/journal.pone.0119231 (PMC4373820; doi:10.1371/journal.pone.0119231)
Supplement: S1 Table — (DOCX) [file pone.0119231.s003.docx]

**S1 Table.** Adaptive-kernel density estimates with *href* for the smoothing parameter of primary and secondary home range and core-use area of 5 feral goats in Pōhakuloa Training Area on Hawai‘i Island, 2010-2011.

| GOAT ID | Primary (P)  Home Range | | Secondary (S)  Home Range | | Distance between 95% P and S Range | Distance between 50%  P and S Range |
| --- | --- | --- | --- | --- | --- | --- |
|  | 50% | 95% | 50% | 95% |  |  |
| M1 | 1.78 | 12.32 | 2.70 | 14.63 | 8.03 | 8.59 |
| M3 | 3.42 | 18.24 | 1.93 | 9.58 | 9.18 | 9.39 |
| M5 | 1.35 | 6.96 | 0.74 | 7.27 | 9.14 | 9.11 |
| F1 | 1.78 | 12.32 | 1.84 | 8.68 | 5.75 | 6.02 |
| F3 | 2.91 | 16.44 | 1.84 | 7.80 | 6.05 | 7.44 |
| **Mean** | **2.25** | **13.26** | **1.81** | **9.59** | **7.63** | **8.11** |
| StDev | 0.87 | 4.37 | 0.70 | 2.95 | 1.65 | 1.39 |
